# Climate‐Related Disaster Adversity and PTSD‐Related Outcomes Among Adolescents in Coastal Bangladesh: A Propensity Score Overlap‐Weighted Analysis

**DOI:** 10.1002/brb3.71894

**Published:** 2026-11-09

**Authors:** Firoj Al Mamun, Mohammed A. Mamun, Maysa Mohamed Rabea Abdelall, Johurul Islam, Mohammad Muhit

**Affiliations:** ^1^ CHINTA Research Bangladesh Dhaka Bangladesh; ^2^ Department of Public Health University of South Asia Dhaka Bangladesh; ^3^ Department of Physical Sport Sciences College of Sport Sciences and Physical Activity Princess Nourah Bint Abdulrahman University Riyadh Saudi Arabia

**Keywords:** adolescents, complex post‐traumatic stress disorder, disaster adversity, post‐traumatic stress disorder (PTSD)

## Abstract

**Background:**

Climate‐related disasters are important environmental stressors for adolescent mental health, particularly in low‐ and middle‐income countries. However, evidence regarding post‐traumatic stress disorder (PTSD) and complex PTSD (CPTSD) among adolescents experiencing cumulative disaster adversity remains limited. This study examined associations between disaster adversity and PTSD‐related outcomes among adolescents in coastal Bangladesh.

**Methods:**

A cross‐sectional school‐based survey included 1697 adolescents from Grades 6–8 across three coastal upazilas between October and November 2025. Disaster adversity was assessed using an eight‐item cumulative index, with ≥4 adversities defining elevated adversity. PTSD‐related outcomes were assessed using the Child and Adolescent Trauma Screen‐2. Physical activity and resilient coping were examined as exploratory correlates. Associations were examined using covariate‐adjusted regression and propensity score overlap weighting with school‐clustered robust inference.

**Results:**

Elevated disaster adversity was associated with higher DSM‐5 PTSD symptom severity (*β* = 5.21, 95% confidence interval [CI] 0.33–10.09). In overlap‐weighted analyses, elevated adversity was associated with a higher prevalence of probable PTSD on the basis of the ≥25 cutoff (prevalence ratio [PR] = 2.00, 95% CI 1.14–3.52), probable International Classification of Diseases, 11th Revision (ICD‐11) PTSD (PR = 2.20, 95% CI 1.10–4.38), and probable ICD‐11 CPTSD (PR = 3.00, 95% CI 1.04–8.67). The estimate for probable DSM‐5 PTSD was PR = 2.01 (95% CI 0.99–4.06), whereas no association was observed with functional impairment (PR = 1.02, 95% CI 0.95–1.10). Physical activity was positively associated only with PTSD symptom severity (*β* = 2.37, 95% CI 0.39–4.36); resilient coping was positively associated with PTSD symptom severity and several probable PTSD outcomes. PTSD‐related symptom burden was higher at greater levels of cumulative adversity, with little evidence of nonlinearity.

**Conclusions:**

Greater cumulative disaster adversity was cross‐sectionally associated with higher PTSD symptom severity and several probable PTSD‐related outcomes. These findings support consideration of adolescent mental health within disaster preparedness and school‐based psychosocial support, whereas longitudinal studies are needed to establish temporal relationships.

## Graphical abstract

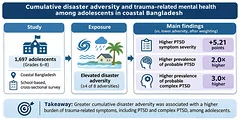

## 1 Introduction

Climate‐related disasters are increasingly recognized as major threats to child and adolescent mental health, particularly in low‐ and middle‐income countries (LMICs) that experience recurrent environmental crises and limited psychosocial support infrastructure (Charlson et al. 2021; Cianconi et al. 2020). Adolescents are especially vulnerable to the psychological consequences of disasters because of ongoing neurodevelopmental, emotional, and social transitions that may heighten sensitivity to trauma and environmental stressors (Pfefferbaum et al. 2016; Sisk and Gee 2022). Exposure to disasters such as cyclones, flooding, displacement, livelihood loss, and educational disruption has been associated with elevated risks of depression, anxiety, psychological distress, and post‐traumatic stress disorder (PTSD) among young people (Charlson et al. 2021; Olk et al. 2025; Rubens et al. 2018).

PTSD is one of the most important truama‐related mental health outcomes following potentiall traumatic experience in children and adolescents (Alisic et al. 2014). Beyond conventional PTSD, recent diagnostic frameworks have highlighted the importance of complex PTSD (CPTSD), particularly in the context of prolonged, cumulative, or repeated adversity (World Health Organization 2025). The International Classification of Diseases, 11th Revision (ICD‐11), distinguishes CPTSD from PTSD by the additional presence of disturbances in self‐organization (DSO), including affect dysregulation, negative self‐concept, and interpersonal difficulties (Cloitre et al. 2018; Sachser et al. 2022). Emerging evidence links natural disasters to mental health problems, while broader childhood adversities have been associated with both PTSD and CPTSD (Heanoy and Brown 2024; Y. Li et al. 2026). However, research examining ICD‐11 PTSD and CPTSD among disaster‐exposed adolescents remains limited, particularly in LMIC settings.

Bangladesh is among the countries most vulnerable to climate‐related disasters globally, with coastal regions facing recurrent cyclones, flooding, salinity intrusion, and storm surges (Uddin et al. 2019). These environmental hazards frequently disrupt housing, livelihoods, education, and social stability, placing adolescents at heightened risk for psychological distress and trauma‐related mental health problems (Patwary et al. 2024). Despite growing recognition of climate‐related mental health challenges, evidence regarding PTSD and CPTSD among adolescents in disaster‐prone regions of Bangladesh remains scarce. Existing studies in Bangladesh have primarily focused on anxiety, depression, or disaster preparedness, with comparatively limited attention to trauma‐specific psychopathology, CPTSD, and associated functional impairment among adolescents (Goldberg et al. 2025; Karim et al. 2024). Bangladesh's high population density, recurrent climate‐related disasters, and limited adolescent mental health infrastructure make it an important context for understanding the psychological consequences of climate‐related adversity among young people in LMIC settings.

In addition to disaster exposure itself, behavioral and psychosocial factors may influence trauma‐related mental health outcomes among adolescents living in disaster‐prone settings (Mesman et al. 2021; Wang et al. 2023). Physical activity has been associated with improved psychological well‐being and reduced mental health symptoms among individuals exposed to traumatic events (Wang et al. 2023). Similarly, resilient coping may serve as a protective psychological resource that facilitates adaptation to stress and adversity (Mesman et al. 2021; Sinclair and Wallston 2004). However, coping responses following chronic disaster exposure may also reflect reactive or compensatory psychological processes among adolescents experiencing elevated distress. Understanding the roles of physical activity and resilient coping may provide important insights into potentially modifiable behavioral and psychosocial factors associated with trauma‐related mental health outcomes among disaster‐exposed adolescents.

Many previous disaster mental health studies have relied on conventional regression approaches that may be vulnerable to residual imbalance between exposed and comparison groups. Propensity score overlap weighting is an analytic approach for improving covariate balance between comparison groups with respect to measured covariates (F. Li et al. 2018; F. Li and Thomas 2019). However, although this approach improves comparability between exposure groups, it cannot establish temporal ordering, infer causality, or eliminate bias arising from unmeasured confounding. This method has rarely been applied in adolescent disaster mental health research conducted in climate‐vulnerable resource‐constrained settings. From a developmental psychopathology perspective, adolescence represents a sensitive period during which repeated exposure to environmental adversity may influence emotional regulation, self‐concept, interpersonal functioning, and stress‐response systems (Sisk and Gee 2022). Although PTSD has been widely examined following disasters, considerably less is known about the relationship between cumulative disaster adversity and ICD‐11 CPTSD among adolescents, particularly in climate‐vulnerable LMIC settings. Understanding whether disaster adversity is associated with broader trauma‐related disturbances beyond core PTSD symptoms may help inform trauma‐informed interventions and mental health policies for young people exposed to recurrent climate‐related disasters.

Therefore, the present study aimed to examine the associations between disaster adversity and PTSD‐related outcomes among adolescents living in disaster‐prone coastal regions of Bangladesh. Specifically, we investigated associations between elevated disaster adversity and PTSD symptom severity, probable DSM‐5 PTSD, probable ICD‐11 PTSD, probable ICD‐11 CPTSD, probable PTSD based on symptom cutoff scores, and functional impairment using propensity score overlap‐weighted analyses. We additionally explored the associations of physical activity and resilient coping with PTSD‐related outcomes.

## 2 Methodology

### 2.1 Study Design and Setting

This study utilized data from the BRIGHT Adolescent Study (Bangladesh Research on Integrated Growth, Health, and Transitions), an ongoing school‐based research initiative examining physical and mental health among adolescents in Bangladesh. A previous analysis from the BRIGHT Adolescent Study examined childhood adversity, physical activity, and cardiometabolic vulnerability (Al Mamun et al. 2026). Data were collected between October and November 2025 from secondary school students in three upazilas (sub‐districts) of Barguna District—Amtali, Taltoli, and Barguna Sadar—located in the southern coastal region of Bangladesh. These coastal upazilas are among the most climate‐vulnerable regions in Bangladesh and are frequently exposed to cyclones, flooding, storm surges, salinity intrusion, and other climate‐related hazards. Recurrent environmental disasters contribute to socioeconomic vulnerability and disruptions in education, livelihoods, and healthcare access, making this setting particularly relevant for investigating adolescent mental health in a disaster‐prone context. Barguna District was purposively selected due to its high exposure to recurrent climate‐related disasters; therefore, the study was not designed to provide a population‐representative sample of adolescents in Barguna District or coastal Bangladesh.

### 2.2 Participants and Sampling

A list of eligible secondary schools and madrasahs was obtained from the district education office. All institutions located in the most disaster‐prone areas of the selected upazilas were approached, and 20 institutions agreed to participate in the study. A school‐based cluster sampling approach was employed, with each participating institution serving as a recruitment cluster. Within each institution, all students enrolled in grades 6, 7, and 8 were invited to participate. These grades correspond to early adolescence and were selected to capture a relatively homogeneous developmental group. The sampling strategy was non‐probability based because the study areas were purposively selected and institutional participation depended on agreement to participate. Furthermore, student participation was limited to eligible school‐attending adolescents who were present during data collection and provided the required assent and parental or guardian consent. Accordingly, the resulting sample should not be considered representative of all adolescents in Barguna District or coastal Bangladesh. Detailed counts of institutions declining participation and of eligible students who were absent, declined participation, did not obtain parental or guardian consent, or were otherwise not enrolled were not systematically recorded during field implementation. Individual‐level characteristics of nonparticipants were also not collected.

### 2.3 Inclusion and Exclusion Criteria

Students were eligible if they were enrolled in grades 6, 7, or 8 in a participating school or madrasah. Participation required attendance during data collection, parental or guardian consent, and student assent. Students with severe physical, visual, hearing, or cognitive impairments that prevented meaningful participation in the classroom‐based survey were excluded. A total of 1697 adolescents were included in the present analytic sample. All variables required for the primary and extended analyses were complete for these participants. No participants were excluded from these analyses because of missing data.

### 2.4 Data Collection Process

Prior to data collection, formal permission was obtained from relevant school authorities, including headteachers and class teachers. Written informed consent was obtained from parents or legal guardians, and participating students provided assent before enrollment in the study. Data were collected in classroom settings using a structured self‐administered questionnaire under the supervision of trained research assistants and field investigators. All research staff received standardized training on study procedures, ethical considerations, confidentiality, and questionnaire administration to ensure consistency across study sites. Participants completed the questionnaire independently, although clarification was provided when necessary. Confidentiality and anonymity were maintained throughout the data collection process. The questionnaire was administered in Bangla. Measures were translated from English into Bangla using forward translation followed by independent back translation into English. The translated and back‐translated versions were reviewed by bilingual experts to assess semantic, conceptual, and contextual equivalence, and discrepancies were resolved before finalizing the Bangla questionnaire. A pilot study involving 30 students was conducted to assess the clarity, readability, and contextual appropriateness of the questionnaire. Minor revisions were made on the basis of participant feedback prior to the main survey, and pilot data were not included in the final analyses. The pilot assessment was conducted to evaluate comprehensibility and contextual appropriateness and did not constitute diagnostic validation of the Child and Adolescent Trauma Screen‐2 (CATS‐2) cutoff or diagnostic criteria.

### 2.5 Measures

#### 2.5.1 Disaster Adversity

Disaster adversity was assessed using a cumulative disaster‐related adversity index derived from adolescents’ experiences of disaster‐related household, educational, and social impacts. Participants were first asked whether they had experienced any of six major climate‐related disasters (cyclone, flood, river erosion, storm surge, salinity intrusion, or drought) during the previous 5 years. They also identified the most recent disaster experienced and reported whether it had occurred within the past 6 months, 6–12 months, 1–2 years, or more than 2 years before the survey. Participants were asked whether their household experienced house damage or loss, crop or livestock loss, drinking water contamination, school closure, household income or livelihood loss, displacement to a shelter or another location, or the loss of a family member or friend following disasters. These items were coded dichotomously (0 = no, 1 = yes). Educational disruption due to disasters was additionally assessed according to the duration of school interruption and categorized as no interruption, less than 1 week, 1–4 weeks, or more than 1 month.

The cumulative disaster adversity score ranging from 0 to 8 was calculated by summing eight binary indicators:, house damage, crop or livestock loss, drinking water contamination, school closure, household income or livelihood loss, displacement, loss of a family member or friend, and any disaster‐related interruption to education. School closure and educational disruption were treated as separate indicators because school closure reflects the occurrence of disaster‐related closure, whereas educational disruption captures the duration of interruption to the participant's education. Higher scores indicated greater disaster adversity burden. The disaster adversity index therefore represents cumulative disaster‐related experiences occurring over varying time intervals rather than exposure to a single disaster event, and the interval between disaster occurrence and the October–November 2025 assessment differed across participants.

For primary analyses, the cumulative score was dichotomized at ≥4 adversities to identify adolescents reporting a relatively high cumulative adversity burden. This threshold was treated as an operational rather than clinically validated cutoff; therefore, the continuous adversity score and alternative thresholds of ≥3 and ≥5 adversities were examined in sensitivity analyses to assess the robustness of findings to the exposure definition.

#### 2.5.2 Post‐Traumatic Stress Symptoms and Probable PTSD

Post‐traumatic stress symptoms were assessed using the CATS‐2, a self‐report screening instrument designed to assess potentially traumatic events, post‐traumatic stress symptoms, and functional impairment according to DSM‐5 and ICD‐11 criteria among children and adolescents aged 7–17 years (Sachser et al. 2022). The CATS‐2 includes a checklist of potentially traumatic events and 25 symptom items assessing trauma‐related symptoms experienced during the past 4 weeks. The life‐event checklist assessed natural‐disaster exposure as well as other potentially traumatic or stressful experiences. Participants could report multiple life events and were not instructed to anchor their PTSD symptom responses to a specific disaster‐related index event. Accordingly, the PTSD‐related outcomes examined in this study cannot be attributed specifically to disaster exposure.

Symptom items are rated on a 4‐point Likert scale ranging from 0 (“never”) to 3 (“almost always”), with higher scores indicating greater symptom severity. The DSM‐5 PTSD severity score was calculated by summing items corresponding to DSM‐5 PTSD symptom criteria (range: 0–60). Probable PTSD was additionally defined using a symptom severity cutoff score of ≥25. Probable DSM‐5 PTSD, probable ICD‐11 PTSD, and probable ICD‐11 CPTSD were identified using the respective DSM‐5 and ICD‐11 diagnostic criteria using the CATS‐2, where symptoms rated as “often” or “almost always” were considered clinically present. The cumulative disaster adversity index was examined as an exposure measure and was not used to establish that individual adversity components satisfied DSM‐5 Criterion A. Potentially traumatic experiences were assessed separately using the CATS‐2 life‐event checklist; however, participants could endorse multiple events, and no single index traumatic event was identified for attribution of PTSD symptoms. Some components, such as educational disruption or livelihood loss, may represent disaster‐related stressors without independently constituting a Criterion A traumatic event. Functional impairment was assessed using five dichotomous items asking whether the problems assessed in the CATS‐2 interfered with getting along with others, hobbies or fun, school or work, family relationships, and general happiness. Responses were coded as 0 = no and 1 = yes. Consistent with the primary operational definition used in this study, any functional impairment was defined as endorsement of impairment in at least one of the five domains; thus, a positive response in a single domain was sufficient for classification. The number of impaired domains was additionally summed (range, 0–5) for sensitivity analyses.

The CATS‐2 has demonstrated good psychometric properties in child and adolescent populations (Sachser et al. 2022). The ≥25 cutoff and DSM‐5/ICD‐11 symptom‐based diagnostic criteria were applied according to the established CATS‐2 scoring procedures but have not been locally validated against clinician‐administered diagnostic interviews among Bangladeshi adolescents. Accordingly, categorical outcomes are described as probable rather than confirmed PTSD or CPTSD. In the present sample, internal consistency was high for the DSM‐5 PTSD symptom scale (Cronbach's *α* = 0.93; McDonald's *ω* = 0.94). Reliability was also acceptable to high for the ICD‐11 PTSD symptom scale (*α* = 0.80; *ω* = 0.86), DSO symptoms (*α* = 0.82; *ω* = 0.87), and the overall ICD‐11 CPTSD symptom scale (*α* = 0.89; *ω* = 0.90). For the two‐item DSO domains, Spearman–Brown coefficients were 0.68 for affect dysregulation, 0.65 for negative self‐concept, and 0.57 for disturbed relationships. These reliability estimates characterize internal consistency and should not be interpreted as evidence of diagnostic validity in Bangladeshi adolescents.

#### 2.5.3 Physical Activity

Adolescents’ physical activity was assessed using the Physical Activity Questionnaire for Adolescents (PAQ‐A), a widely used self‐administered 7‐day recall instrument designed to evaluate general moderate‐to‐vigorous physical activity levels among adolescents (Kowalski et al. 1997). Items are rated on a 5‐point Likert scale, with higher scores indicating greater frequency and intensity of physical activity participation. The overall PAQ‐A score was calculated by averaging eight activity‐related items, resulting in a composite score ranging from 1 to 5, where higher scores reflect greater levels of habitual physical activity. Internal consistency of the PAQ‐A in the present sample was acceptable (Cronbach's *α* = 0.73).

#### 2.5.4 Resilient Coping

Resilient coping was assessed using the Brief Resilient Coping Scale (BRCS), a brief self‐report instrument designed to measure adaptive coping tendencies in response to stress (Sinclair and Wallston 2004). The BRCS consists of four items rated on a 5‐point Likert scale ranging from 1 (“does not describe me at all”) to 5 (“describes me very well”). Total scores are calculated by summing item responses, resulting in a score range of 4–20, with higher scores indicating greater resilient coping capacity. Internal consistency of the BRCS in the present sample was good (Cronbach's *α* = 0.84).

#### 2.5.5 Sociodemographic Characteristics

Sociodemographic variables included age, gender, grade level, monthly family income, parental education, family type, house type, upazila, and institution type. Family income was categorized as <25,000 BDT and ≥25,000 BDT per month. Parental education was categorized as primary or less versus secondary or higher. Family type was categorized as nuclear or joint family, and house type was categorized as tin‐shed or brick‐built housing. Upazila was categorized as Barguna Sadar, Amtali, or Taltoli, and institution type as secondary school or madrasah.

### 2.6 Ethical Considerations

All procedures performed in this study involving human participants were conducted in accordance with the ethical standards of the relevant institutional and national research committees and with the Declaration of Helsinki and its subsequent amendments. Ethical approval was obtained from the Institutional Review Board of CHINTA Research Bangladesh [Ref: CHINTA/IRB/07‐2025/8(6)].

### 2.7 Conceptual Framework

Covariate selection and analytical modeling were guided by the conceptual framework shown in Figure 1. The primary estimand was the covariate‐adjusted total cross‐sectional association between cumulative disaster adversity and PTSD‐related outcomes. Physical activity and resilient coping were examined only as exploratory correlates in extended models because their temporal positions relative to disaster adversity and PTSD‐related outcomes could not be established.

**FIGURE 1:**
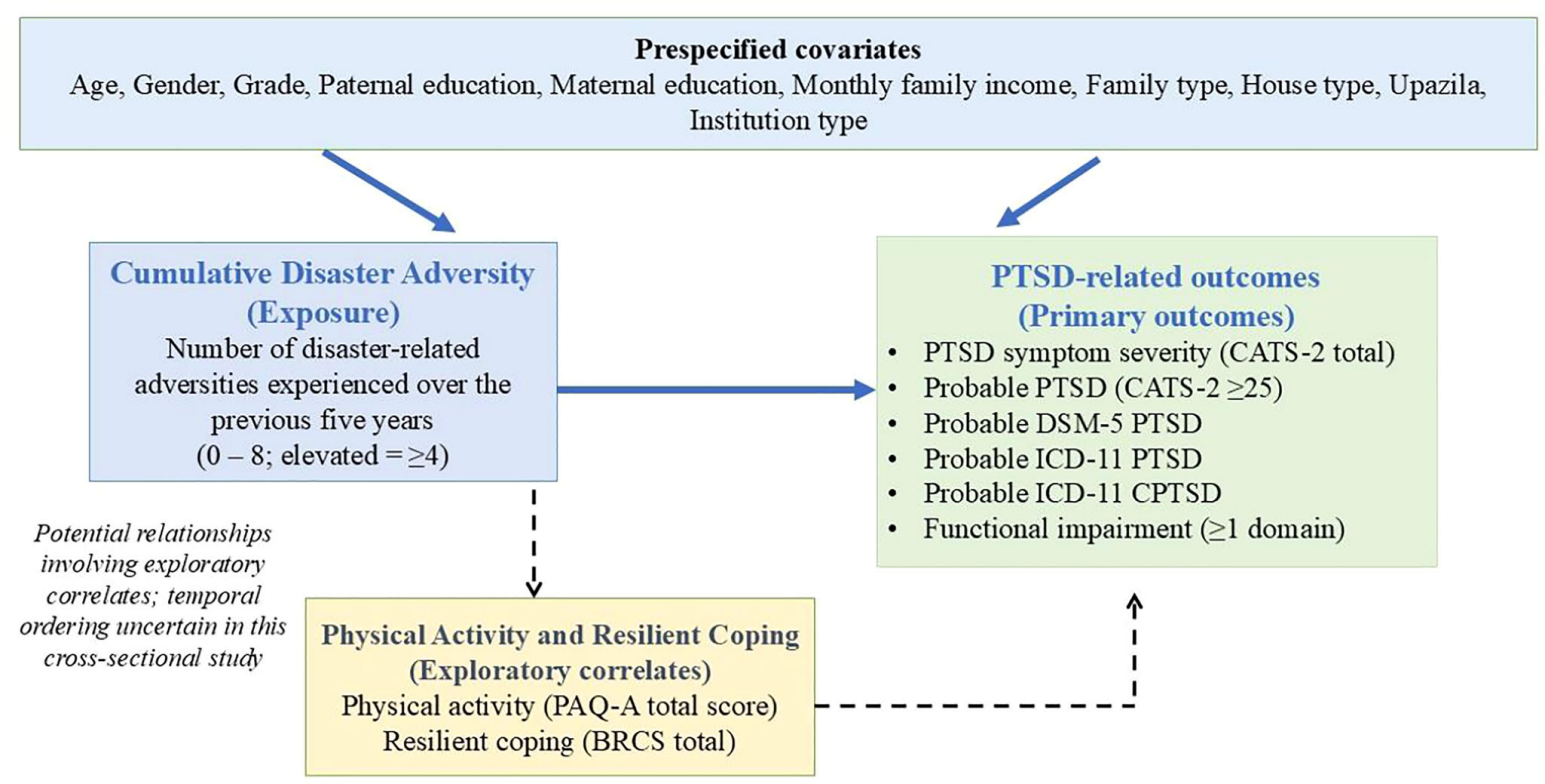
Conceptual framework for covariate selection and analytical modeling. Prespecified covariates were included in the primary adjustment set, whereas physical activity and resilient coping were examined as exploratory correlates because their temporal positions could not be established. Solid arrows represent the primary conceptual associations, and dashed arrows represent temporally uncertain relationships; arrows do not imply causal relationships. BRCS, Brief Resilient Coping Scale; CATS‐2, Child and Adolescent Trauma Screen‐2; ICD‐11, International Classification of Diseases, 11th Revision; PAQ‐A, Physical Activity Questionnaire for Adolescents; PTSD, post‐traumatic stress disorder.

### 2.8 Statistical Analysis

Data analyses were conducted using R statistical software (version 4.4.1). Descriptive statistics were presented as mean and standard deviation (SD) for continuous variables and frequencies with percentages for categorical variables. Group differences according to disaster adversity status were assessed using Welch's two‐sample *t*‐tests for continuous variables and Pearson's *χ* ^2^ tests for categorical variables.

PTSD symptom severity was assessed using the total score of the CATS‐2 on the basis of DSM‐5 criteria. Probable PTSD outcomes were examined using multiple operationalizations, including a symptom cutoff score (CATS‐2 ≥ 25), the DSM‐5 PTSD diagnostic criteria, the ICD‐11 PTSD diagnostic criteria, and the ICD‐11 CPTSD diagnostic criteria. Functional impairment was defined as the presence of impairment in at least one domain of functioning.

School‐level clustering was examined using intraclass correlation coefficients (ICCs) from random‐intercept models. The ICC for continuous DSM‐5 PTSD symptom severity was estimated using a linear mixed model, whereas ICCs for binary outcomes were estimated on the latent scale from logistic mixed models using a Level‐1 residual variance of *π* ^2^/3. The ICC was 0.21 for DSM‐5 PTSD symptom severity and ranged from 0.20 to 0.30 across the binary PTSD‐related outcomes (probable PTSD based on the CATS‐2 cutoff ≥25, 0.26; probable DSM‐5 PTSD, 0.20; probable ICD‐11 PTSD, 0.21; probable ICD‐11 CPTSD, 0.30; and any functional impairment, 0.25). Accordingly, all regression analyses used CR2 cluster‐robust variance estimators with schools as the clustering unit and Satterthwaite small‐sample degrees‐of‐freedom corrections. For the overlap‐weighted models, estimated overlap weights were treated as fixed in the outcome‐stage variance calculations.

To improve covariate balance between low and elevated disaster adversity with respect to prespecified measured covariates, propensity score overlap weighting was implemented using the *WeightIt* package, with generalized linear modeling and the average treatment effect in the overlap population (ATO) estimand. The ATO estimand emphasizes adolescents with covariate profiles having greater overlap in the predicted probability of low versus elevated disaster adversity and therefore pertains to the covariate‐overlap population rather than representing a population‐average association for the full study sample. Propensity scores were estimated based on age, gender, grade, family income, parental education, family type, house type, upazila, and institution type. Covariate balance before and after weighting was evaluated using standardized mean differences (SMDs), with absolute SMD values <0.10 considered indicative of acceptable balance. Variance ratios were also examined for continuous covariates, and balance was additionally evaluated for a quadratic age term and an age‐by‐gender interaction. Propensity‐score distributions were examined by adversity group to assess common support, and overlap‐weight distributions were summarized using the minimum, maximum, selected percentiles, and effective sample size. Post‐weighting balance was interpreted as comparability with respect to the measured covariates rather than evidence that residual confounding had been eliminated. Although overlap weighting improves comparability between exposure groups with respect to measured covariates, it cannot establish causal relationships, determine temporal ordering, or account for unmeasured confounding.

Primary overlap‐weighted outcome models included disaster adversity status as the exposure, with adjustment for the prespecified covariates achieved through the propensity‐score overlap weights. Extended weighted models additionally included physical activity (PAQ‐A total score) and resilient coping (BRCS total score), which were treated as exploratory correlates rather than variables required for estimation of the primary association. For continuous DSM‐5 PTSD symptom severity, overlap‐weighted linear regression was used, and associations were expressed as regression coefficients (*β*). For binary PTSD‐related outcomes, overlap‐weighted logistic regression was used, and associations were expressed as prevalence odds ratios (ORs) with 95% confidence intervals (CIs). Because several outcomes were common, these ORs were treated as complementary association measures rather than risk or incidence measures. To provide prevalence‐based relative association measures that are more directly interpretable for common binary outcomes, overlap‐weighted modified Poisson regression with a log link was additionally used to estimate adjusted prevalence ratios (PRs) and 95% CIs for both primary and extended models.

Adjusted marginal probabilities for each binary PTSD‐related outcome according to disaster adversity status were estimated from the primary overlap‐weighted logistic models. CIs for predicted probabilities were calculated on the logit scale and back‐transformed to the probability scale. Adjusted prevalence differences were estimated on the response scale and expressed as absolute percentage‐point differences between adolescents with elevated and low disaster adversity, using school‐clustered CR2 variance estimation.

Several complementary analyses were conducted to evaluate the robustness and functional form of the observed associations. First, the cumulative disaster adversity score (range, 0–8) was modeled as a continuous exposure. Linear regression was used for DSM‐5 PTSD symptom severity and modified Poisson regression with a log link for binary PTSD‐related outcomes, adjusting for the prespecified covariates. Quadratic terms for cumulative adversity were additionally examined to assess potential departures from linearity. Covariate‐adjusted mean DSM‐5 PTSD symptom severity across the full adversity‐score range was estimated from the linear model and displayed graphically. Second, sensitivity analyses examined alternative thresholds for defining elevated disaster adversity (≥3 and ≥5 adversities) in addition to the primary threshold of ≥4 adversities. Third, the individual disaster‐related adversity components were examined separately in relation to selected PTSD‐related outcomes; Benjamini–Hochberg false discovery rate (FDR) correction was applied separately within each outcome to account for multiple comparisons. Fourth, analyses were repeated among adolescents who reported natural‐disaster exposure on the CATS‐2 life‐event checklist. Finally, because any functional impairment was highly prevalent, sensitivity analyses examined more restrictive impairment thresholds (impairment in ≥2 and ≥3 domains) and the number of impaired functional domains. All complementary regression analyses accounted for school‐level clustering using CR2 variance estimation.

All variables required for the primary and extended regression models were complete for the 1697 adolescents; therefore, these analyses included the full analytic sample, and multiple imputation was not required. Statistical tests were two‐sided, with *p* < 0.05 considered statistically significant. For the adversity‐component analyses, both nominal and FDR‐adjusted p‐values were reported.

## 3 Results

### 3.1 Participant Characteristics

A total of 1697 adolescents were included, of whom 454 (26.8%) had elevated disaster adversity (≥4 adversities) and 1243 (73.2%) had low disaster adversity. The mean age was 13.05 years (SD = 1.10), and 64.3% were girls. Participants with elevated disaster adversity more frequently had lower monthly family income, lower maternal education, and tin‐shed housing and differed by upazila and institution type (all *p* < 0.05) (Table 1).

**TABLE 1:** Participant characteristics according to cumulative disaster adversity status (*N* = 1697).

Characteristic	Overall *N* = 1697	Low adversity *N* = 1243	Elevated adversity *N* = 454	*p* value
Age (years), mean (SD)	13.05 (1.10)	13.06 (1.11)	13.04 (1.09)	0.715
Gender, *n* (%)				0.764
Boys	606 (35.7)	447 (36.0)	159 (35.0)	
Girls	1091 (64.3)	796 (64.0)	295 (65.0)	
Grade, *n* (%)				0.890
Grade 6	590 (34.8)	428 (34.4)	162 (35.7)	
Grade 7	548 (32.3)	403 (32.4)	145 (31.9)	
Grade 8	559 (32.9)	412 (33.1)	147 (32.4)	
Monthly family income (BDT), *n* (%)				<0.001
<25,000	1142 (67.3)	807 (64.9)	335 (73.8)	
≥25,000	555 (32.7)	436 (35.1)	119 (26.2)	
Father's education, *n* (%)				0.371
Primary or less	981 (57.8)	710 (57.1)	271 (59.7)	
Secondary or higher	716 (42.2)	533 (42.9)	183 (40.3)	
Mother's education, *n* (%)				0.045
Primary or less	898 (52.9)	639 (51.4)	259 (57.0)	
Secondary or higher	799 (47.1)	604 (48.6)	195 (43.0)	
Family type, *n* (%)				0.098
Nuclear	1335 (78.7)	965 (77.6)	370 (81.5)	
Joint	362 (21.3)	278 (22.4)	84 (18.5)	
House type, *n* (%)				0.042
Tin shed	1448 (85.3)	1047 (84.2)	401 (88.3)	
Brick built	249 (14.7)	196 (15.8)	53 (11.7)	
Upazila, *n* (%)				<0.001
Barguna Sadar	424 (25.0)	315 (25.3)	109 (24.0)	
Amtali	373 (22.0)	180 (14.5)	193 (42.5)	
Taltoli	900 (53.0)	748 (60.2)	152 (33.5)	
Institution type, *n* (%)				<0.001
Secondary school	1451 (85.5)	1094 (88.0)	357 (78.6)	
Madrasah	246 (14.5)	149 (12.0)	97 (21.4)	
Physical activity score, mean (SD)	1.94 (0.62)	1.94 (0.63)	1.95 (0.58)	0.559
Brief Resilient Coping Scale score, mean (SD)	11.10 (4.50)	10.85 (4.60)	11.81 (4.14)	<0.001
DSM‐5 PTSD symptom severity score, mean (SD)	14.61 (12.25)	12.63 (11.53)	20.03 (12.56)	<0.001
Probable PTSD (CATS‐2 cutoff ≥25), *n* (%)				<0.001
No	1294 (76.3)	1030 (82.9)	264 (58.1)	
Yes	403 (23.7)	213 (17.1)	190 (41.9)	
Probable DSM‐5 PTSD, *n* (%)				<0.001
No	1456 (85.8)	1119 (90.0)	337 (74.2)	
Yes	241 (14.2)	124 (10.0)	117 (25.8)	
Probable ICD‐11 PTSD, *n* (%)				<0.001
No	1533 (90.3)	1164 (93.6)	369 (81.3)	
Yes	164 (9.7)	79 (6.4)	85 (18.7)	
Probable ICD‐11 CPTSD, *n* (%)				<0.001
No	1616 (95.2)	1212 (97.5)	404 (89.0)	
Yes	81 (4.8)	31 (2.5)	50 (11.0)	
Any functional impairment, *n* (%)				0.002
No	205 (12.1)	169 (13.6)	36 (7.9)	
Yes	1492 (87.9)	1074 (86.4)	418 (92.1)	

*Note*: Values are presented as mean (SD) or *n* (%). Elevated disaster adversity was defined as exposure to ≥4 disaster‐related adversities, whereas low disaster adversity was defined as exposure to 0–3 adversities. *p* values were calculated using Welch's two‐sample t‐test for continuous variables and Pearson's *χ* ^2^ test for categorical variables.

Abbreviations: CATS‐2, Child and Adolescent Trauma Screen‐2; CPTSD, complex post‐traumatic stress disorder; ICD‐11, International Classification of Diseases, 11th Revision; PTSD, post‐traumatic stress disorder; SD, standard deviation.

Mean DSM‐5 PTSD symptom severity was higher among adolescents with elevated than low disaster adversity (20.03 vs. 12.63, *p* < 0.001). Probable PTSD based on the CATS‐2 cutoff ≥25 (41.9% vs. 17.1%), probable DSM‐5 PTSD (25.8% vs. 10.0%), probable ICD‐11 PTSD (18.7% vs. 6.4%), probable ICD‐11 CPTSD (11.0% vs. 2.5%), and any functional impairment (92.1% vs. 86.4%) were also more prevalent among adolescents with elevated disaster adversity (Table 1).

### 3.2 Distribution of Disaster Exposures and Disaster‐Related Adversities

Cyclone (97.5%) and flood (64.1%) were the most commonly reported disaster exposures, whereas educational disruption (88.5%), school closure (66.1%), and house damage or destruction (41.6%) were the most frequently reported disaster‐related adversities. The cumulative disaster adversity score had a mean of 2.78 (SD = 1.72) and a median of 2.00 (IQR = 2.00–4.00). Overall, 41.8%, 26.8%, and 16.9% of adolescents experienced ≥3, ≥4, and ≥5 adversities, respectively (Table S1).

### 3.3 Associations Between Elevated Disaster Adversity and PTSD‐Related Outcomes

In the primary overlap‐weighted linear model, elevated disaster adversity was associated with higher DSM‐5 PTSD symptom severity (*β* = 5.21, 95% CI 0.33–10.09; *p* = 0.038). The association remained similar in the extended weighted model, additionally including physical activity and resilient coping as exploratory correlates (*β* = 5.00, 95% CI 0.27–9.72; *p* = 0.040) (Table 2).

**TABLE 2:** Overlap‐weighted linear and logistic associations between elevated disaster adversity and PTSD‐related outcomes with school‐clustered robust inference (*N* = 1697).

Outcome	Model	Predictor	Effect measure	Estimate (95% CI)	*p* value
DSM‐5 PTSD symptom severity	Primary	Elevated disaster adversity	*β*	5.21 (0.33, 10.09)	0.038
	Extended	Elevated disaster adversity	*β*	5.00 (0.27, 9.72)	0.040
		Physical activity (PAQ‐A total)	*β*	2.37 (0.39, 4.36)	0.024
		Brief resilient coping (BRCS total)	*β*	0.32 (0.08, 0.57)	0.015
Probable PTSD (CATS‐2 cutoff ≥25)	Primary	Elevated disaster adversity	OR	2.57 (1.06, 6.26)	0.039
	Extended	Elevated disaster adversity	OR	2.54 (1.05, 6.10)	0.039
		Physical activity (PAQ‐A total)	OR	1.28 (0.91, 1.81)	0.145
		Brief resilient coping (BRCS total)	OR	1.04 (0.99, 1.10)	0.095
Probable DSM‐5 PTSD	Primary	Elevated disaster adversity	OR	2.29 (0.94, 5.61)	0.066
	Extended	Elevated disaster adversity	OR	2.24 (0.92, 5.42)	0.071
		Physical activity (PAQ‐A total)	OR	1.03 (0.74, 1.43)	0.850
		Brief resilient coping (BRCS total)	OR	1.07 (1.01, 1.13)	0.026
Probable ICD‐11 PTSD	Primary	Elevated disaster adversity	OR	2.41 (1.07, 5.47)	0.037
	Extended	Elevated disaster adversity	OR	2.35 (1.05, 5.27)	0.039
		Physical activity (PAQ‐A total)	OR	1.11 (0.76, 1.63)	0.551
		Brief resilient coping (BRCS total)	OR	1.07 (1.02, 1.12)	0.012
Probable ICD‐11 CPTSD	Primary	Elevated disaster adversity	OR	3.19 (1.00, 10.13)	0.050
	Extended	Elevated disaster adversity	OR	3.10 (0.99, 9.72)	0.052
		Physical activity (PAQ‐A total)	OR	0.94 (0.52, 1.69)	0.807
		Brief resilient coping (BRCS total)	OR	1.08 (1.03, 1.13)	0.002
Any functional impairment	Primary	Elevated disaster adversity	OR	1.22 (0.61, 2.45)	0.546
	Extended	Elevated disaster adversity	OR	1.19 (0.59, 2.39)	0.597
		Physical activity (PAQ‐A total)	OR	2.04 (0.91, 4.58)	0.079
		Brief resilient coping (BRCS total)	OR	1.02 (0.97, 1.08)	0.371

*Note*: Estimates are presented as *β* coefficients for the continuous outcome (DSM‐5 PTSD symptom severity) and prevalence odds ratios (ORs) for binary outcomes, with 95% confidence intervals. Propensity scores were estimated using age, gender, grade, monthly family income, paternal education, maternal education, family type, house type, upazila, and institution type, and these covariates were incorporated through overlap weighting. Primary weighted outcome models included elevated disaster adversity as the predictor. Extended weighted models additionally included physical activity (PAQ‐A total score) and resilient coping (BRCS total score) as exploratory correlates. Standard errors and confidence intervals were estimated using CR2 cluster‐robust variance estimators, accounting for clustering within 20 schools with Satterthwaite small‐sample degrees‐of‐freedom corrections. Estimated overlap weights were treated as fixed in the outcome‐stage variance calculations. All analyses were based on the complete analytic sample (*N* = 1697).

Abbreviations: BRCS, Brief Resilient Coping Scale; CATS‐2, Child and Adolescent Trauma Screen‐2; CI, confidence interval; CPTSD, complex post‐traumatic stress disorder; ICD‐11, International Classification of Diseases, 11th Revision; OR, prevalence odds ratio; PAQ‐A, Physical Activity Questionnaire for Adolescents; PTSD, post‐traumatic stress disorder; *β*, regression coefficient.

For binary outcomes, elevated disaster adversity was associated with higher odds of probable PTSD on the basis of the CATS‐2 cutoff ≥25 in both the primary (OR = 2.57, 95% CI 1.06–6.26; *p* = 0.039) and extended (OR = 2.54, 95% CI 1.05–6.10; *p* = 0.039) models. Similarly, higher odds of probable ICD‐11 PTSD were observed in the primary (OR = 2.41, 95% CI 1.07–5.47; *p* = 0.037) and extended (OR = 2.35, 95% CI 1.05–5.27; *p* = 0.039) models. For probable ICD‐11 CPTSD, the association was at the conventional significance threshold in the primary model (OR = 3.19, 95% CI 1.00–10.13; *p* = 0.050) and was similar in magnitude in the extended model (OR = 3.10, 95% CI 0.99–9.72; *p* = 0.052).

For probable DSM‐5 PTSD, the estimates were OR = 2.29 (95% CI 0.94–5.61; *p* = 0.066) in the primary model and OR = 2.24 (95% CI 0.92–5.42; *p* = 0.071) in the extended model. Elevated disaster adversity was not associated with any functional impairment in either the primary (OR = 1.22, 95% CI 0.61–2.45; *p* = 0.546) or extended (OR = 1.19, 95% CI 0.59–2.39; *p* = 0.597) model (Table 2).

### 3.4 Overlap‐Weighted Associations and Adjusted Absolute Differences

Following propensity‐score overlap weighting, all prespecified covariates had absolute SMD < 0.10. Variance ratios for continuous covariates were also within the prespecified balance criterion. Balance was additionally observed for the nonlinear age term and age‐by‐gender interaction. Overlap weights ranged from 0.065 to 0.660 in the low‐adversity group and from 0.325 to 0.928 in the elevated‐adversity group; the corresponding effective sample sizes were 967 and 423. Propensity‐score distributions showed substantial common support between the adversity groups (Table S2; Figures S1–S2).

In the overlap‐weighted analyses, elevated disaster adversity was associated with a higher prevalence of probable PTSD on the basis of the CATS‐2 cutoff ≥25 in both the primary (PR = 2.00, 95% CI 1.14–3.52; *p* = 0.020) and extended (PR = 1.97, 95% CI 1.13–3.41; *p* = 0.020) models. Similar associations were observed for probable ICD‐11 PTSD (primary: PR = 2.20, 95% CI 1.10–4.38; *p* = 0.029; extended: PR = 2.14, 95% CI 1.09–4.20; *p* = 0.031) and probable ICD‐11 CPTSD (primary: PR = 3.00, 95% CI 1.04–8.67; *p* = 0.043; extended: PR = 2.91, 95% CI 1.03–8.23; *p* = 0.045). For probable DSM‐5 PTSD, the corresponding estimates were PR = 2.01 (95% CI 0.99–4.06; *p* = 0.052) and PR = 1.95 (95% CI 0.98–3.89; *p* = 0.056), respectively. Elevated disaster adversity was not associated with any functional impairment in either model (Table 3).

**TABLE 3:** Overlap‐weighted associations between elevated disaster adversity and PTSD‐related outcomes (*N* = 1697).

Outcome	Model	Adjusted prevalence ratio (95% CI)	*p* value
Probable PTSD (CATS‐2 cutoff ≥25)	Primary	2.00 (1.14, 3.52)	0.020
	Extended	1.97 (1.13, 3.41)	0.020
Probable DSM‐5 PTSD	Primary	2.01 (0.99, 4.06)	0.052
	Extended	1.95 (0.98, 3.89)	0.056
Probable ICD‐11 PTSD	Primary	2.20 (1.10, 4.38)	0.029
	Extended	2.14 (1.09, 4.20)	0.031
Probable ICD‐11 CPTSD	Primary	3.00 (1.04, 8.67)	0.043
	Extended	2.91 (1.03, 8.23)	0.045
Any functional impairment	Primary	1.02 (0.95, 1.10)	0.545
	Extended	1.02 (0.95, 1.10)	0.574

*Note*: Propensity scores were estimated using age, gender, grade, monthly family income, paternal education, maternal education, family type, house type, upazila, and institution type, and these covariates were incorporated through overlap weighting. Primary weighted outcome models included elevated disaster adversity as the predictor, whereas extended weighted models additionally included physical activity (PAQ‐A total score) and resilient coping (BRCS total score) as exploratory correlates. Adjusted prevalence ratios were estimated using overlap‐weighted modified Poisson regression with CR2 cluster‐robust variance estimation accounting for clustering within 20 schools and Satterthwaite small‐sample corrections. The estimated overlap weights were treated as fixed in the outcome‐stage variance calculations. All analyses were based on 1697 adolescents.

Abbreviations: CATS‐2, Child and Adolescent Trauma Screen‐2; CI, confidence interval; CPTSD, complex post‐traumatic stress disorder; ICD‐11, International Classification of Diseases, 11th Revision; PR, prevalence ratio; PTSD, post‐traumatic stress disorder.

On the absolute scale, the adjusted prevalence of probable PTSD based on the CATS‐2 cutoff ≥25 was 18.28% (95% CI 14.26–23.12) among adolescents with low disaster adversity and 36.53% (95% CI 19.40–57.92) among those with elevated disaster adversity, corresponding to an adjusted prevalence difference of 18.25 percentage points (95% CI −0.65 to 37.16; *p* = 0.058). The adjusted prevalence differences were 11.08 percentage points (95% CI −2.72 to 24.89; *p* = 0.116) for probable DSM‐5 PTSD, 8.37 percentage points (95% CI −1.66 to 18.39; *p* = 0.102) for probable ICD‐11 PTSD, 5.59 percentage points (95% CI −2.62 to 13.81; *p* = 0.182) for probable ICD‐11 CPTSD, and 1.89 percentage points (95% CI −4.05 to 7.83; *p* = 0.533) for any functional impairment (Table 4).

**TABLE 4:** Adjusted marginal probabilities and prevalence differences for PTSD‐related outcomes according to disaster adversity status.

Outcome	Adjusted marginal probabilities		Adjusted prevalence difference, percentage points (95% CI)	*p* value
	Low adversity, % (95% CI)	Elevated adversity, % (95% CI)		
Probable PTSD (CATS‐2 cutoff ≥25)	18.28 (14.26, 23.12)	36.53 (19.40, 57.92)	18.25 (−0.65, 37.16)	0.058
Probable DSM‐5 PTSD	11.01 (8.01, 14.96)	22.10 (10.91, 39.65)	11.08 (−2.72, 24.89)	0.116
Probable ICD‐11 PTSD	6.99 (5.04, 9.61)	15.36 (7.26, 29.61)	8.37 (−1.66, 18.39)	0.102
Probable ICD‐11 CPTSD	2.79 (2.00, 3.88)	8.39 (2.95, 21.63)	5.59 (−2.62, 13.81)	0.182
Any functional impairment	88.41 (80.52, 93.36)	90.30 (82.59, 94.81)	1.89 (−4.05, 7.83)	0.533

*Note*: Adjusted marginal probabilities were estimated from the primary overlap‐weighted logistic regression models. Confidence intervals for adjusted probabilities were calculated on the logit scale and back‐transformed to the probability scale. Adjusted prevalence differences represent the absolute percentage‐point difference in predicted prevalence between adolescents with elevated and low disaster adversity. Variance estimation used CR2 cluster‐robust standard errors to account for clustering within schools. p values correspond to the adjusted prevalence‐difference contrasts.

Abbreviations: CI, confidence interval; CPTSD, complex post‐traumatic stress disorder; PTSD, post‐traumatic stress disorder; ICD‐11, International Classification of Diseases, 11th Revision.

### 3.5 Continuous Associations With Cumulative Disaster Adversity

When cumulative disaster adversity was examined as a continuous score, each one‐unit higher adversity score was associated with greater DSM‐5 PTSD symptom severity (*β* = 1.48, 95% CI 0.42–2.54; p = 0.010). Higher cumulative adversity was also associated with a higher prevalence of probable PTSD on the basis of the CATS‐2 cutoff ≥25 (PR = 1.20, 95% CI 1.08–1.33; p = 0.003), probable DSM‐5 PTSD (PR = 1.19, 95% CI 1.06–1.34; p = 0.008), probable ICD‐11 PTSD (PR = 1.19, 95% CI 1.04–1.36; p = 0.015), and probable ICD‐11 CPTSD (PR = 1.25, 95% CI 1.03–1.53; p = 0.031). Cumulative disaster adversity was not associated with any functional impairment (PR = 1.01, 95% CI 0.99–1.03; p = 0.291) (Table S3).

There was no evidence of departure from linearity for any PTSD‐related outcome, with all quadratic terms being nonsignificant (Table S3). Consistent with these findings, adjusted mean DSM‐5 PTSD symptom severity increased across the cumulative disaster adversity score, from 10.49 (95% CI 7.75–13.22) at a score of 0 to 22.34 (95% CI 16.35–28.34) at a score of 8 (Figure S3).

### 3.6 Sensitivity Analyses Using Alternative Disaster‐Adversity Thresholds

The associations were further examined using alternative thresholds of ≥3 and ≥5 disaster‐related adversities, alongside the primary threshold of ≥4. For DSM‐5 PTSD symptom severity, elevated adversity was associated with higher symptom severity across all three thresholds: ≥3 adversities (*β* = 3.40, 95% CI 0.63–6.16; p = 0.021), ≥4 adversities (*β* = 5.21, 95% CI 1.21–9.20; p = 0.015), and ≥5 adversities (*β* = 6.57, 95% CI 1.34–11.80; p = 0.019).

For the binary PTSD‐related outcomes, the magnitude of the associations was generally larger at higher adversity thresholds. At the ≥5‐adversity threshold, higher prevalence was observed for probable PTSD on the basis of the CATS‐2 cutoff ≥25 (PR = 2.22, 95% CI 1.48–3.33; p = 0.001), probable DSM‐5 PTSD (PR = 2.15, 95% CI 1.34–3.47; p = 0.005), probable ICD‐11 PTSD (PR = 2.19, 95% CI 1.25–3.86; p = 0.012), and probable ICD‐11 CPTSD (PR = 2.91, 95% CI 1.45–5.85; p = 0.007). In contrast, no association with any functional impairment was observed across the alternative thresholds. Overall, these analyses indicated that the pattern of associations was not dependent on the primary ≥4‐adversity definition (Table S4).

### 3.7 Exploratory Associations of Individual Disaster‐Adversity Components With PTSD‐Related Outcomes

Exploratory analyses of individual disaster‐adversity components showed that several components were associated with PTSD‐related outcomes, although the pattern varied across outcomes. After correction for multiple comparisons, drinking‐water contamination (*β* = 6.12, 95% CI 1.98–10.26; FDR‐p = 0.042) and income or livelihood loss (*β* = 5.22, 95% CI 1.48–8.96; FDR‐p = 0.042) were associated with higher DSM‐5 PTSD symptom severity. For probable PTSD based on the CATS‐2 cutoff ≥25, associations remained after FDR correction for crop or livestock loss (PR = 1.49, 95% CI 1.10–2.02; FDR‐p = 0.029), drinking‐water contamination (PR = 2.13, 95% CI 1.47–3.10; FDR‐p = 0.010), income or livelihood loss (PR = 1.77, 95% CI 1.22–2.58; FDR‐p = 0.026), loss of a family member or friend (PR = 1.84, 95% CI 1.14–3.00; FDR‐p = 0.039), and displacement (PR = 1.68, 95% CI 1.16–2.43; FDR‐p = 0.029).

Although several individual adversity components were associated with probable ICD‐11 CPTSD before correction for multiple comparisons, none remained statistically significant after FDR correction. Educational disruption and school closure were not associated with the examined PTSD‐related outcomes after FDR correction (Table S5).

### 3.8 Natural‐Disaster‐Exposed Subgroup Analysis

In the subgroup of adolescents who reported a natural disaster as a traumatic life event (N = 957), overlap‐weighted analyses showed that elevated disaster adversity was associated with higher DSM‐5 PTSD symptom severity (*β* = 5.31, 95% CI 0.10–10.51; p = 0.046). Elevated disaster adversity was also associated with a higher prevalence of probable PTSD on the basis of the CATS‐2 cutoff ≥25 (PR = 1.92, 95% CI 1.11–3.32; p = 0.023), probable DSM‐5 PTSD (PR = 2.03, 95% CI 1.05–3.92; p = 0.037), probable ICD‐11 PTSD (PR = 2.45, 95% CI 1.34–4.48; p = 0.007), and probable ICD‐11 CPTSD (PR = 4.93, 95% CI 1.44–16.88; p = 0.015). No association was observed with any functional impairment (PR = 0.99, 95% CI 0.94–1.04; p = 0.736). These subgroup findings were broadly consistent with the pattern observed in the full analytic sample (Table S6). Other potentially traumatic experiences were also commonly reported (Table S7), and CATS‐2 symptom responses were not anchored to the natural disaster as an index event. These subgroup findings should not be interpreted as estimates of disaster‐attributable PTSD.

### 3.9 Functional‐Impairment Sensitivity Analyses

Functional impairment was common across the five individual domains, ranging from 74.1% for general happiness to 80.6% for getting along with others. Under the primary definition, 87.9% of adolescents reported impairment in at least one domain. Overall, 85.4% reported impairment in at least two domains, 81.7% in at least three domains, and 60.6% in all five domains. The mean number of impaired domains was 3.88 (Table S8).

Given the high prevalence of any functional impairment in the sample, sensitivity analyses examined more stringent impairment thresholds and the number of impaired functional domains. Elevated disaster adversity was not associated with functional impairment when defined as impairment in at least two domains (PR = 1.03, 95% CI 0.97–1.08; p = 0.351) or at least three domains (PR = 1.01, 95% CI 0.95–1.08; p = 0.780). Similarly, elevated disaster adversity was not associated with the number of impaired functional domains (count ratio = 1.00, 95% CI 0.94–1.07; p = 0.916) (Table S8). Thus, the null association was consistent across the primary and more restrictive functional‐impairment definitions.

## 4 Discussion

The present study examined associations between cumulative disaster adversity and PTSD‐related outcomes among adolescents in disaster‐prone coastal regions of Bangladesh using complementary covariate‐adjusted and overlap‐weighted analyses. Elevated disaster adversity was associated with higher DSM‐5 PTSD symptom severity and a higher prevalence of several probable PTSD‐related outcomes. In the overlap‐weighted analyses, adolescents with elevated disaster adversity had approximately twice the prevalence of probable PTSD based on the CATS‐2 cutoff ≥25 and probable ICD‐11 PTSD, and approximately three times the prevalence of probable ICD‐11 CPTSD, compared with adolescents with low disaster adversity. The estimate for probable DSM‐5 PTSD was similar in direction and magnitude, although its confidence interval included the null. In contrast, elevated disaster adversity was not associated with functional impairment. The findings were generally consistent across primary and extended models and several complementary analyses, indicating a cross‐sectional pattern of greater PTSD‐related symptom burden with higher cumulative disaster adversity.

The continuous adversity‐score analyses further supported this pattern. Each additional disaster‐related adversity was associated with higher DSM‐5 PTSD symptom severity and a higher prevalence of probable PTSD on the basis of the CATS‐2 cutoff ≥25, probable DSM‐5 PTSD, probable ICD‐11 PTSD, and probable ICD‐11 CPTSD. There was little evidence of departure from linearity across these outcomes, suggesting that PTSD‐related symptom burden generally increased with accumulating disaster adversity rather than being confined to adolescents meeting the primary threshold of ≥4 adversities. Consistent with this finding, sensitivity analyses using alternative thresholds of ≥3 and ≥5 adversities showed associations in the same general direction, although estimates varied in magnitude and precision across thresholds and outcomes. Together, these findings suggest that variation across the full cumulative adversity score provides information beyond the primary dichotomous classification in understanding PTSD‐related outcomes in this population.

The findings align with previous evidence linking trauma exposure to PTSD symptoms and natural disasters to broader psychological distress among children and adolescents (Alisic et al. 2014; Meltzer et al. 2021; Olk et al. 2025; Rubens et al. 2018). Disaster‐related adversities may co‐occur across several domains of adolescents’ lives, including housing, household livelihoods, access to safe drinking water, education, displacement, and bereavement. In the present sample, educational disruption and school closure were particularly common, whereas substantial proportions also reported house damage, crop or livestock loss, drinking‐water contamination, and livelihood loss. Such cumulative experiences may coincide with disruptions in family stability, schooling, social connectedness, and perceived safety (Karim et al. 2024; Patwary et al. 2024; Uddin et al. 2019). The higher PTSD symptom severity observed among adolescents reporting greater cumulative adversity is therefore consistent with a broader literature linking the accumulation of stressful and potentially traumatic experiences with greater psychological symptom burden. However, disaster adversity and PTSD‐related outcomes were assessed cross‐sectionally, therefore, the present findings cannot establish temporal ordering or determine whether the reported disaster adversities contributed causally to the observed PTSD‐related symptoms.

Exploratory analyses of the individual adversity components provided additional insight into the pattern of associations across specific disaster‐related experiences. After correction for multiple comparisons, drinking‐water contamination and loss of household income or livelihood were associated with higher DSM‐5 PTSD symptom severity and a higher prevalence of probable PTSD on the basis of the CATS‐2 cutoff ≥25. Crop or livestock loss, loss of a family member or friend, and displacement were also associated with probable PTSD on the basis of the ≥25 cutoff after FDR correction, whereas associations for several other individual components were less consistent. No component remained associated with probable ICD‐11 CPTSD after FDR correction. These findings suggest that the observed cumulative association may reflect contributions from multiple forms of disaster‐related disruption rather than a single adversity component. Given the exploratory nature of these component‐specific analyses and the potential for correlated exposures, however, individual component estimates should not be interpreted as identifying specific causal effects or ranking particular adversities by importance.

Although the relative association estimate for probable ICD‐11 CPTSD was numerically larger than those for the other binary outcomes, this should not be interpreted as evidence that disaster adversity was more strongly associated with CPTSD than with PTSD. The CPTSD estimate was based on relatively few cases and consequently had a wide confidence interval. Moreover, relative association measures, such as ORs and PRs, should not be directly compared across outcomes with different baseline prevalences. On the absolute scale, elevated adversity corresponded to estimated prevalence differences of 18.25 percentage points for probable PTSD based on the CATS‐2 cutoff ≥25, 11.08 percentage points for probable DSM‐5 PTSD, 8.37 percentage points for probable ICD‐11 PTSD, and 5.59 percentage points for probable ICD‐11 CPTSD, although the confidence intervals for these differences included the null. Accordingly, the findings are better interpreted as showing a broadly consistent pattern of higher PTSD‐related symptom burden across several operationalizations of probable PTSD, rather than evidence of a comparatively stronger association with CPTSD.

The absence of an adjusted association with functional impairment warrants particular consideration. Although functional impairment was more prevalent descriptively among adolescents with elevated than low disaster adversity, this difference was not evident after covariate adjustment or overlap weighting. Functional impairment was highly prevalent in the overall sample (87.9%), potentially limiting differentiation between adversity groups. Importantly, sensitivity analyses using progressively more restrictive definitions, impairment in at least two or at least three functional domains, also showed no association with elevated disaster adversity, and the number of impaired domains was similarly unrelated to adversity status. Thus, the null finding was not specific to the original definition of any impairment. Impairment was common across all five domains (74.1%–80.6%), and 60.6% of participants endorsed impairment in all five domains. The high prevalence may partly reflect the broad dichotomous assessment and the primary definition, under which endorsement of impairment in a single domain was sufficient for classification. More broadly, considerable heterogeneity has been documented in the domains and scoring approaches used to assess trauma‐related functional impairment in children and adolescents (Vogt et al. 2026). The impairment findings should, therefore, be interpreted separately from the symptom‐based PTSD outcomes and with appropriate caution.

Analyses restricted to adolescents who endorsed natural‐disaster exposure on the CATS‐2 life‐event checklist yielded a pattern broadly consistent with the full‐sample analyses. Within this subgroup, elevated disaster adversity was associated with higher DSM‐5 PTSD symptom severity and a higher prevalence of probable PTSD on the basis of the ≥25 cutoff, probable DSM‐5 PTSD, probable ICD‐11 PTSD, and probable ICD‐11 CPTSD, whereas functional impairment again showed no association. These findings provide additional support for the consistency of the observed cross‐sectional associations. However, because participants could report other potentially traumatic life events and CATS‐2 symptom responses were not explicitly anchored to the reported natural disaster as the index traumatic event, the subgroup analysis reduces but does not eliminate uncertainty regarding symptom attribution. Accordingly, these findings should be interpreted as associations between disaster adversity and PTSD‐related outcomes among adolescents reporting natural‐disaster exposure rather than as estimates of disaster‐attributable PTSD.

Physical activity and resilient coping were included only in the extended models as exploratory correlates rather than prespecified baseline confounders. Their inclusion produced little change in the estimated associations between disaster adversity and PTSD‐related outcomes, suggesting that the adversity estimates were not materially changed by statistical adjustment for these characteristics. Resilient coping was positively associated with PTSD symptom severity and several probable PTSD outcomes in the extended regression models. Given the cross‐sectional design, this finding should not be interpreted as evidence that resilient coping increases PTSD‐related symptoms. Greater coping engagement may occur in response to greater psychological burden, and the temporal ordering of coping and PTSD‐related symptoms cannot be established from these data. Accordingly, the BRCS findings are best regarded as exploratory cross‐sectional correlates rather than evidence of either a protective or harmful role of resilient coping.

Physical activity showed a positive association with DSM‐5 PTSD symptom severity in the extended model but was not associated with the probable PTSD outcomes, whereas its relationship with functional impairment did not reach conventional statistical significance. Although physical activity has been associated with better mental health among trauma‐exposed individuals (Wang et al. 2023), the present cross‐sectional findings do not establish the direction or nature of this relationship in adolescents experiencing disaster adversity. Physical activity could reflect multiple behavioral, social, or contextual processes that were not captured in the present analyses. Given the inconsistent pattern across outcomes and the uncertain temporal ordering of physical activity relative to PTSD‐related symptoms, these findings should be interpreted cautiously and should not be taken as evidence that physical activity increases PTSD‐related psychopathology.

### 4.1 Implications

Adolescence represents a sensitive developmental period during which trauma‐related difficulties may have long‐term implications for emotional, social, and academic functioning. The present findings therefore highlight the importance of considering trauma‐related mental health needs among adolescents living in disaster‐prone settings, particularly those reporting multiple disaster‐related adversities. In climate‐vulnerable and resource‐constrained communities where access to specialist child and adolescent mental health services may be limited, schools could provide accessible settings for identifying and supporting adolescents experiencing substantial trauma‐related symptoms. Recent evidence indicates that school‐based interventions can reduce PTSD symptoms among children and adolescents following natural disasters (Laksmita et al. 2026). Rather than supporting routine diagnostic screening on the basis of these cross‐sectional findings alone, the results suggest a need to evaluate contextually appropriate approaches to early identification, referral, and trauma‐informed psychosocial support within school and community settings.

The observed graded pattern further suggests that attention should not be restricted to adolescents crossing a single threshold of disaster adversity. Adolescents experiencing accumulating disruptions across housing, livelihoods, water security, education, displacement, and bereavement may represent an important group for further assessment and support. In Bangladesh, where specialist child and adolescent mental health resources are limited, integration of mental health considerations into school health programs, disaster preparedness, and post‐disaster recovery services may provide opportunities to strengthen support for disaster‐affected adolescents. However, the effectiveness, feasibility, cultural acceptability, and potential unintended consequences of screening or intervention approaches require evaluation before widespread implementation. As climate‐related disasters pose substantial challenges for vulnerable communities, understanding the mental health needs of adolescents experiencing cumulative disaster‐related adversity remains an important public mental health priority.

Methodologically, the study demonstrates the value of examining disaster adversity using complementary analytical approaches rather than relying exclusively on a single dichotomous exposure definition or effect measure. In addition to conventional covariate‐adjusted models, overlap weighting achieved acceptable covariate balance according to the prespecified balance criterion, and PRs and absolute prevalence differences complemented odds‐ratio estimates for the binary outcomes. Continuous adversity‐score analyses, alternative adversity thresholds, component‐specific analyses, and analyses restricted to adolescents reporting natural‐disaster exposure provided additional assessments of the consistency and form of the observed associations. These complementary analyses strengthen the descriptive epidemiological characterization of the associations but do not overcome the temporal and residual‐confounding limitations inherent in the cross‐sectional design.

### 4.2 Strengths and Limitations

Several limitations should be considered when interpreting the findings. First, disaster adversity, physical activity, resilient coping, and PTSD‐related outcomes were measured cross‐sectionally; therefore, the temporal ordering and causal relationships cannot be established. Reverse causation remains possible, and propensity score overlap weighting cannot determine whether disaster adversity preceded PTSD‐related outcomes or account for unmeasured confounding. Furthermore, the disaster adversity index captured cumulative experiences over the previous 5 years; therefore, the interval between individual disaster‐related experiences and PTSD‐related symptom assessment varied across participants. Although analyses restricted to adolescents endorsing natural‐disaster exposure on the CATS‐2 life‐event checklist yielded broadly similar findings, PTSD symptoms were not explicitly anchored to the reported natural disaster as the index traumatic event. Participants also reported other potentially traumatic life events, further limiting disaster‐specific symptom attribution. Consequently, the results should be interpreted as associations between disaster adversity and PTSD‐related outcomes rather than as evidence of disaster‐attributable PTSD. Longitudinal studies with clearly defined exposure timing, index traumatic events, and repeated symptom assessments are needed to characterize temporal relationships and trajectories of PTSD‐related symptoms following disaster exposure.

Second, disaster‐related adversities and PTSD‐related outcomes were based on self‐report and may be affected by recall, reporting, or social desirability biases. The cumulative adversity index assigned equal weight to eight heterogeneous disaster‐related experiences and did not capture the severity, duration, recurrence, geographic intensity, or precise timing of each adversity. Moreover, some components may be conceptually related, for example, school closure and educational disruption, although they captured distinct aspects of disaster‐related educational impact. The index should, therefore, be interpreted as a pragmatic measure of cumulative reported disaster‐related adversity rather than a validated measure of disaster exposure intensity.

Third, although the CATS‐2 provides established DSM‐5 and ICD‐11 symptom‐based criteria, probable PTSD and CPTSD were identified using a self‐report screening instrument rather than clinician‐administered diagnostic interviews. Accordingly, these outcomes represent probable rather than confirmed diagnoses. The CATS‐2 ≥ 25 cutoff and DSM‐5/ICD‐11 diagnostic criteria have not been locally validated against clinician‐administered diagnoses among Bangladeshi adolescents. Therefore, despite the observed internal consistency of the relevant symptom scales and the use of translation, back‐translation, expert review, and pilot testing, the diagnostic accuracy and cultural equivalence of these classifications in this population remain uncertain, and outcome misclassification is possible. The functional‐impairment measure also warrants caution because 87.9% of participants endorsed impairment in at least one domain. Although sensitivity analyses using impairment in ≥2 and ≥3 domains and the number of impaired domains produced similarly null associations with elevated disaster adversity, the high overall prevalence may indicate limited discriminatory capacity of the dichotomous impairment items in this population.

Fourth, although the propensity score model incorporated a range of measured sociodemographic, geographic, and institutional covariates, residual confounding by unmeasured or incompletely measured factors remains possible. Potentially relevant factors such as prior trauma exposure, baseline mental health, family psychiatric history, parental mental health, household conflict, social support, disability or chronic illness, objective disaster severity, disaster frequency and recency, and other school‐ or community‐level characteristics were not comprehensively accounted for. Because disaster adversity captured experiences over the previous 5 years, the temporal ordering of current family income and house type relative to disaster adversity could not be established, and these characteristics may themselves have been influenced by previous disaster experiences. They were, therefore, treated as measured sociodemographic covariates rather than established pre‐exposure confounders. Physical activity and resilient coping were not treated as baseline confounders because their temporal positions relative to disaster adversity and PTSD‐related symptoms could not be established. Instead, they were included only in extended models as exploratory correlates. Therefore, overlap weighting should not be interpreted as eliminating confounding or producing causal estimates.

Fifth, the overlap‐weighted estimates correspond to the average treatment effect in the overlap population (ATO) and therefore pertain most directly to adolescents whose measured characteristics were compatible with either low or elevated disaster adversity. They should not necessarily be interpreted as population‐average associations for all adolescents in the study setting, particularly those with covariate profiles strongly predictive of one adversity group. The observed common support and post‐weighting covariate balance support comparability of the adversity groups with respect to the measured covariates within the weighted population but do not exclude residual confounding by unmeasured factors. In addition, estimated overlap weights were treated as fixed in the outcome‐stage variance calculations; therefore, uncertainty arising from propensity‐score estimation was not additionally propagated into the reported standard errors.

Sixth, participants were school‐attending adolescents recruited from selected secondary schools and madrasahs in Barguna Sadar, Amtali, and Taltoli upazilas of Barguna District. The sampling strategy was non‐probability based, and participation depended on institutional agreement, school attendance during data collection, and parental or guardian consent and adolescent assent. Adolescents who were absent, displaced, out of school, or otherwise unable to participate may have differed systematically in disaster adversity and PTSD‐related outcomes from those included, potentially introducing selection bias. Detailed recruitment counts for institutional and student nonparticipation and individual‐level characteristics of nonparticipants were not systematically recorded, precluding reconstruction of a complete participant‐flow diagram and comparison of participants with nonparticipants. The findings therefore should not be generalized to all adolescents in coastal Bangladesh, particularly those not attending school, or necessarily to adolescents in other geographic, socioeconomic, or cultural settings. Future studies incorporating community‐based samples and adolescents outside the formal education system would help assess the generalizability of these findings.

Despite these limitations, the present study has several strengths. It included a large sample of 1697 school‐attending adolescents from a highly climate‐vulnerable coastal setting and evaluated PTSD‐related outcomes using multiple DSM‐5‐ and ICD‐11‐based operationalizations, including symptom severity, probable PTSD, probable CPTSD, and functional impairment. The study also characterized cumulative disaster adversity both categorically and continuously rather than relying on a single exposure specification. School‐level clustering was explicitly accounted for using CR2 cluster‐robust variance estimation with Satterthwaite small‐sample degrees‐of‐freedom corrections, which was important given the observed school‐level ICCs and the inclusion of only 20 participating schools. Propensity score overlap weighting achieved acceptable balance across the measured sociodemographic covariates, including nonlinear and interaction terms examined in the balance diagnostics.

Additional strengths include reporting PRs alongside ORs for common binary outcomes, estimating adjusted absolute prevalence differences, evaluating continuous and nonlinear associations, examining alternative adversity thresholds, conducting exploratory component‐specific analyses with FDR correction, restricting analyses to adolescents reporting natural‐disaster exposure, and evaluating alternative definitions of functional impairment. Together, these complementary analyses provide a more comprehensive assessment of the consistency and form of the cross‐sectional associations while avoiding reliance on any single exposure threshold, outcome definition, or effect measure.

## 5 Conclusion

In conclusion, greater cumulative disaster adversity was associated with higher PTSD symptom severity and a higher prevalence of several probable PTSD‐related outcomes among school‐attending adolescents in disaster‐prone coastal regions of Bangladesh. These associations were observed across DSM‐5‐ and ICD‐11‐based PTSD operationalizations and were generally supported by overlap‐weighted and sensitivity analyses. Continuous analyses further indicated that PTSD‐related symptom burden generally increased with accumulating disaster adversity, with little evidence of departure from linearity. In contrast, disaster adversity was not associated with functional impairment after adjustment, including when more restrictive impairment definitions were examined.

These findings indicate that cumulative disaster‐related adversity is an important cross‐sectional correlate of PTSD‐related symptoms among adolescents in this climate‐vulnerable setting, but they do not establish temporal or causal relationships or demonstrate that the PTSD‐related outcomes were attributable specifically to disaster exposure. The findings should, therefore, be interpreted as a broadly consistent pattern across several PTSD‐related outcomes rather than evidence that disaster adversity was more strongly associated with any particular PTSD or CPTSD outcome. The findings support consideration of adolescents’ mental health needs within school health, disaster preparedness, and post‐disaster psychosocial support initiatives, whereas further research is needed to determine the effectiveness and appropriateness of specific intervention approaches. Longitudinal research with clearly characterized disaster exposures and repeated mental health assessments is needed to establish temporal relationships, examine trajectories of PTSD and CPTSD, and identify potentially modifiable protective and vulnerability factors among adolescents experiencing recurrent disaster‐related adversity.

## Author Contributions

**Firoj Al Mamun**: conceptualization, methodology, formal analysis, data curation, visualization, validation, writing – original draft, writing – review and editing. **Mohammed A. Mamun**: conceptualization, methodology, writing – review and editing. **Maysa Mohamed Rabea Abdelall**: writing – review and editing, visualization, validation, data interpretation. **Johurul Islam**: data curation, investigation, writing – review and editing. **Mohammad Muhit**: conceptualization, supervision, visualization, validation, writing – review and editing. All authors critically reviewed the manuscript, approved the final version for publication, and agree to be accountable for all aspects of the work.

## Funding

Dr Maysa Mohamed Rabea Abdelall is currently receiving funding support from the Princess Nourah Bint Abdulrahman University Researchers Supporting Project (Number PNURSP2026R802), Princess Nourah Bint Abdulrahman University, Riyadh, Saudi Arabia.

## Conflicts of Interest

The authors declare no conflicts of interest.

## Supporting information




**Supplementary material**: brb371894‐sup‐0001‐SuppMat.docx

## Data Availability

The dataset is available from the corresponding author upon reasonable request.
