# Supplementary material for: Climate‐Related Disaster Adversity and PTSD‐Related Outcomes Among Adolescents in Coastal Bangladesh: A Propensity Score Overlap‐Weighted Analysis
Source: Brain Behav. 2026 Nov 9;16(11):e71894. doi: 10.1002/brb3.71894 (PMC13746987; doi:10.1002/brb3.71894)
Supplement: Supplementary file 1 [file BRB3-16-e71894-s001.docx]

Supplementary material for

**Climate-related disaster adversity and PTSD-related outcomes among adolescents in coastal Bangladesh: a propensity score overlap-weighted analysis**

Firoj Al Mamun ^1,2^, Mohammed A Mamun ^1,2^, Maysa Mohamed Rabea Abdelall ^3^, Johurul Islam ^2^, Mohammad Muhit ^2^

1. CHINTA Research Bangladesh, Dhaka 1342, Bangladesh
2. Department of Public Health, University of South Asia, Dhaka 1348, Bangladesh
3. Department of Physical Sport Sciences, College of Sport Sciences and Physical Activity, Princess Nourah bint Abdulrahman University, Riyadh 11671, Saudi Arabia

**Correspondence:** Firoj Al Mamun, CHINTA Research Bangladesh, Dhaka 1342, Bangladesh and University of South Asia, Dhaka 1348, Bangladesh. Email: firojphiju@gmail.com; Contact: +8801902430861

Supplementary Table S1. Distribution of disaster exposures, disaster-related adversities, and cumulative disaster adversity among adolescents (N = 1,697)

| **Characteristic** | **n** | **%** |
| --- | --- | --- |
| Disaster exposure during the past 5 years |  |  |
| Cyclone | 1,655 | 97.50 |
| Flood | 1,087 | 64.10 |
| River erosion | 422 | 24.90 |
| Salinity | 406 | 23.90 |
| Storm surge | 356 | 21.00 |
| Drought | 268 | 15.80 |
| Disaster-related adversity components |  |  |
| Educational disruption | 1,502 | 88.50 |
| School closure | 1,121 | 66.10 |
| House damage/destruction | 706 | 41.60 |
| Crop or livestock loss | 469 | 27.60 |
| Drinking-water contamination | 342 | 20.20 |
| Loss of family income/livelihood | 327 | 19.30 |
| Relocation to shelter or another location | 199 | 11.70 |
| Loss of family member or friend | 54 | 3.18 |
| Duration of disaster-related educational disruption |  |  |
| No interruption | 195 | 11.50 |
| Less than one week | 1,233 | 72.70 |
| One to four weeks | 221 | 13.00 |
| More than one month | 48 | 2.83 |
| Cumulative disaster adversity score |  |  |
| 0 | 54 | 3.18 |
| 1 | 283 | 16.70 |
| 2 | 650 | 38.30 |
| 3 | 256 | 15.10 |
| 4 | 167 | 9.84 |
| 5 | 105 | 6.19 |
| 6 | 118 | 6.95 |
| 7 | 44 | 2.59 |
| 8 | 20 | 1.18 |
| **Cumulative disaster adversity score:** mean (SD) = **2.78 (1.72)**; median (IQR) = **2.00 (2.00–4.00)**; range, **0–8**. | | |
| Adversity thresholds |  |  |
| ≥3 adversities | 710 | 41.80 |
| ≥4 adversities (primary elevated-adversity threshold) | 454 | 26.80 |
| ≥5 adversities | 287 | 16.90 |
| **Note:** Values are n (%) unless otherwise indicated. Participants could report exposure to more than one disaster type. The cumulative disaster adversity score was calculated as the sum of eight binary disaster-related adversity components, yielding a possible range of 0–8. Educational disruption was defined as any disaster-related interruption to education; no interruption was coded as absence of educational disruption. The primary analysis defined elevated disaster adversity as ≥4 adversities. Thresholds of ≥3 and ≥5 adversities were examined in sensitivity analyses. Complete adversity-score data were available for all 1,697 participants. | | |

Supplementary Table S2. Propensity-score overlap-weighting diagnostics

| **Panel A. Covariate balance before and after overlap weighting** | | | | |
| --- | --- | --- | --- | --- |
| **Covariate** | **Unweighted SMD** | **Overlap-weighted SMD** | **Unweighted variance ratio** | **Weighted variance ratio** |
| Age | -0.02 | 0.00 | 0.96 | 0.96 |
| Gender: girls | 0.01 | 0.00 | — | — |
| Grade: 6th | 0.01 | 0.00 | — | — |
| Grade: 7th | 0.00 | 0.00 | — | — |
| Grade: 8th | -0.01 | 0.00 | — | — |
| Monthly family income: ≥25,000 BDT | -0.09 | 0.00 | — | — |
| Father education: secondary or higher | -0.03 | 0.00 | — | — |
| Mother education: secondary or higher | -0.06 | 0.00 | — | — |
| Family type: joint | -0.04 | 0.00 | — | — |
| House type: brick-built | -0.04 | 0.00 | — | — |
| Upazila: Barguna Sadar | -0.01 | 0.00 | — | — |
| Upazila: Amtali | 0.28 | 0.00 | — | — |
| Upazila: Taltoli | -0.27 | 0.00 | — | — |
| Institution type: Madrasah | 0.09 | 0.00 | — | — |
| Age² | -0.02 | 0.00 | 0.96 | 0.94 |
| Age × gender | 0.02 | 0.00 | 1.00 | 1.02 |

**Panel B. Overlap-weight diagnostics**

| **Disaster adversity group** | **n** | **Mean (SD)** | **Minimum** | **P1** | **P5** | **P25** | **Median** | **P75** | **P95** | **P99** | **Maximum** | **Effective sample size** |
| --- | --- | --- | --- | --- | --- | --- | --- | --- | --- | --- | --- | --- |
| Low adversity | 1,243 | 0.236 (0.127) | 0.065 | 0.083 | 0.102 | 0.149 | 0.202 | 0.277 | 0.535 | 0.628 | 0.660 | 967 |
| Elevated adversity | 454 | 0.647 (0.174) | 0.325 | 0.344 | 0.377 | 0.478 | 0.693 | 0.794 | 0.872 | 0.911 | 0.928 | 423 |

**Panel C. Propensity-score distribution and common support**

| **Disaster adversity group** | **Minimum** | **P1** | **P5** | **P25** | **Median** | **Mean** | **P75** | **P95** | **P99** | **Maximum** |
| --- | --- | --- | --- | --- | --- | --- | --- | --- | --- | --- |
| Low adversity | 0.06 | 0.08 | 0.10 | 0.15 | 0.20 | 0.24 | 0.28 | 0.54 | 0.63 | 0.66 |
| Elevated adversity | 0.07 | 0.09 | 0.13 | 0.21 | 0.31 | 0.35 | 0.52 | 0.62 | 0.66 | 0.68 |

**Note:** SMD, standardized mean difference; P1, 1st percentile; P5, 5th percentile; P25, 25th percentile; P75, 75th percentile; P95, 95th percentile; P99, 99th percentile. Propensity scores were estimated from age, gender, grade, monthly family income, paternal education, maternal education, family type, house type, upazila, and institution type. Overlap weights targeted the average treatment effect in the overlap population (ATO). Absolute SMDs <0.10 and variance ratios <2 were considered indicative of adequate balance. Post-weighting balance reflects comparability with respect to the measured covariates and should not be interpreted as evidence that residual confounding was eliminated.

**Supplementary Figure S1. Propensity-score distributions and common support according to disaster adversity group.**

**Supplementary Figure S2. Covariate balance before and after propensity-score overlap weighting.**

Supplementary Table S3. Linear and quadratic associations between cumulative disaster adversity and PTSD-related outcomes

| **Outcome** | **Effect measure** | **Linear estimate (95% CI)** | ***p*-value** | **Quadratic estimate (95% CI)** | ***p*-value** |
| --- | --- | --- | --- | --- | --- |
| DSM-5 PTSD symptom severity | β | 1.48 (0.42, 2.54) | 0.010 | 0.13 (−0.15, 0.42) | 0.323 |
| Probable PTSD (CATS-2 cutoff ≥25) | PR | 1.20 (1.08, 1.33) | 0.003 | 1.02 (0.97, 1.07) | 0.360 |
| Probable DSM-5 PTSD | PR | 1.19 (1.06, 1.34) | 0.008 | 1.02 (0.97, 1.07) | 0.495 |
| Probable ICD-11 PTSD | PR | 1.19 (1.04, 1.36) | 0.015 | 1.02 (0.97, 1.07) | 0.463 |
| Probable ICD-11 CPTSD | PR | 1.25 (1.03, 1.53) | 0.031 | 1.01 (0.94, 1.09) | 0.772 |
| Any functional impairment | PR | 1.01 (0.99, 1.03) | 0.291 | 1.00 (0.99, 1.01) | 0.943 |
| **Note:** Linear estimates represent the change associated with each one-unit increase in the cumulative disaster adversity score. β coefficients are reported for continuous PTSD symptom severity, whereas prevalence ratios (PRs) are reported for binary outcomes. Quadratic terms were included to assess departure from linearity. Models were adjusted for the prespecified sociodemographic covariates and used CR2 cluster-robust variance estimation for clustering within 20 schools. All models included N = 1,697. | | | | | |

**Supplementary Figure S3. Dose–response association between cumulative disaster adversity and DSM-5 PTSD symptom severity.**

Supplementary Table S4. Sensitivity analyses using alternative thresholds for cumulative disaster adversity

| **Outcome** | **Effect measure** | **≥3 adversities, estimate (95% CI)** | ***p*-value** | **≥4 adversities, estimate (95% CI)** | ***p*-value** | **≥5 adversities, estimate (95% CI)** | ***p*-value** |
| --- | --- | --- | --- | --- | --- | --- | --- |
| DSM-5 PTSD symptom severity | β | 3.40 (0.63, 6.16) | 0.021 | 5.21 (1.21, 9.20) | 0.015 | 6.57 (1.34, 11.80) | 0.019 |
| Probable PTSD (CATS-2 cutoff ≥25) | PR | 1.48 (0.96, 2.29) | 0.071 | 1.91 (1.22, 2.98) | 0.009 | 2.22 (1.48, 3.33) | 0.001 |
| Probable DSM-5 PTSD | PR | 1.40 (0.79, 2.49) | 0.216 | 1.89 (1.10, 3.26) | 0.026 | 2.15 (1.34, 3.47) | 0.005 |
| Probable ICD-11 PTSD | PR | 1.56 (0.94, 2.59) | 0.081 | 2.01 (1.19, 3.39) | 0.013 | 2.19 (1.25, 3.86) | 0.012 |
| Probable ICD-11 CPTSD | PR | 1.80 (0.73, 4.45) | 0.178 | 2.51 (1.06, 5.93) | 0.038 | 2.91 (1.45, 5.85) | 0.007 |
| Any functional impairment | PR | 1.04 (0.97, 1.10) | 0.242 | 1.02 (0.97, 1.08) | 0.415 | 1.03 (0.94, 1.12) | 0.543 |
| **Note:** β = regression coefficient; PR = prevalence ratio; CI = confidence interval; PTSD = post-traumatic stress disorder; CPTSD = complex post-traumatic stress disorder. Each threshold compares adolescents meeting or exceeding the specified cumulative disaster-adversity threshold with those below that threshold. Models were adjusted for the prespecified sociodemographic covariates and used CR2 cluster-robust variance estimation to account for clustering within 20 schools. Linear regression was used for DSM-5 PTSD symptom severity, whereas Poisson regression with a log link and CR2 robust variance estimation was used to estimate PRs for binary outcomes. All analyses included N = 1,697. | | | | | | | |

Supplementary Table S5. Exploratory associations of individual disaster-adversity components with PTSD-related outcomes

| **Disaster-adversity component** | **PTSD symptom severity, β (95% CI)** | ***p*** | **FDR-*p*** | **Probable PTSD (≥25), PR (95% CI)** | ***p*** | **FDR-*p*** | **Probable ICD-11 CPTSD, PR (95% CI)** | ***p*** | **FDR-*p*** |
| --- | --- | --- | --- | --- | --- | --- | --- | --- | --- |
| Crop or livestock loss | 3.24 (0.57–5.90) | 0.021 | 0.057 | 1.49 (1.10–2.02) | 0.015 | 0.029 | 1.68 (0.92–3.08) | 0.083 | 0.166 |
| Drinking water contamination | 6.12 (1.98–10.26) | 0.008 | 0.042 | 2.13 (1.47–3.10) | 0.001 | 0.010 | 2.51 (1.12–5.58) | 0.029 | 0.114 |
| Educational disruption | 2.22 (−1.06–5.51) | 0.157 | 0.179 | 1.36 (0.86–2.15) | 0.162 | 0.185 | 2.10 (0.68–6.46) | 0.161 | 0.215 |
| House damage or loss | 3.00 (0.15–5.85) | 0.041 | 0.065 | 1.41 (0.91–2.17) | 0.108 | 0.144 | 2.27 (1.03–5.00) | 0.043 | 0.114 |
| Income or livelihood loss | 5.22 (1.48–8.96) | 0.010 | 0.042 | 1.77 (1.22–2.58) | 0.006 | 0.026 | 2.08 (1.05–4.13) | 0.038 | 0.114 |
| Loss of family member or friend | 4.38 (−1.03–9.79) | 0.097 | 0.130 | 1.84 (1.14–3.00) | 0.024 | 0.039 | 1.40 (0.57–3.46) | 0.315 | 0.360 |
| Displacement | 3.87 (0.43–7.31) | 0.031 | 0.063 | 1.68 (1.16–2.43) | 0.012 | 0.029 | 1.42 (0.83–2.43) | 0.161 | 0.215 |
| School closure | 0.78 (−1.28–2.84) | 0.402 | 0.402 | 1.11 (0.77–1.60) | 0.510 | 0.510 | 0.99 (0.48–2.03) | 0.968 | 0.968 |
| **Note:** β = regression coefficient; PR = prevalence ratio; CI = confidence interval; PTSD = post-traumatic stress disorder; CPTSD = complex post-traumatic stress disorder; FDR = false discovery rate. Each disaster-adversity component was examined in a separate model. Linear regression was used for DSM-5 PTSD symptom severity, whereas Poisson regression with a log link was used to estimate prevalence ratios for binary outcomes. All models were adjusted for the prespecified sociodemographic covariates and used CR2 cluster-robust variance estimation to account for clustering within 20 schools. Benjamini–Hochberg FDR-adjusted p-values were calculated separately within each outcome. All models included **N = 1,697**; there were **403 events** for probable PTSD based on the CATS-2 cutoff ≥25 and **81 events** for probable ICD-11 CPTSD. | | | | | | | | | |

Supplementary Table S6. Associations between elevated disaster adversity and PTSD-related outcomes among adolescents reporting natural-disaster exposure on the CATS-2 life-event checklist

| **Outcome** | **Effect measure** | **Estimate (95% CI)** | ***p*-value** | **Analytic N** | **Events, n** |
| --- | --- | --- | --- | --- | --- |
| DSM-5 PTSD symptom severity | β | 5.31 (0.10–10.51) | 0.046 | 957 | — |
| Probable PTSD (CATS-2 cutoff ≥25) | PR | 1.92 (1.11–3.32) | 0.023 | 957 | 294 |
| Probable DSM-5 PTSD | PR | 2.03 (1.05–3.92) | 0.037 | 957 | 167 |
| Probable ICD-11 PTSD | PR | 2.45 (1.34–4.48) | 0.007 | 957 | 118 |
| Probable ICD-11 CPTSD | PR | 4.93 (1.44–16.88) | 0.015 | 957 | 56 |
| Any functional impairment | PR | 0.99 (0.94–1.04) | 0.736 | 957 | 884 |
| **Note:** β = regression coefficient; PR = prevalence ratio; CI = confidence interval; PTSD = post-traumatic stress disorder; CPTSD = complex post-traumatic stress disorder. Analyses were restricted to adolescents who endorsed the CATS-2 life-event item indicating exposure to a serious natural disaster. Within this subgroup, propensity scores for elevated disaster adversity were re-estimated using the prespecified sociodemographic covariates, and overlap weights targeting the overlap population were applied. Linear regression was used for DSM-5 PTSD symptom severity and Poisson regression with a log link for binary outcomes. CR2 cluster-robust variance estimation accounted for clustering within schools. The subgroup included **957 adolescents.** | | | | | |

Supplementary Table S7. CATS-2 life-event exposures reported by adolescents (N = 1,697)

| **CATS-2 life-event exposure** | **Available N** | **n** | **%** |
| --- | --- | --- | --- |
| Serious natural disaster | 1,697 | 957 | 56.4 |
| Serious accident or injury | 1,697 | 729 | 43.0 |
| Witnessed or experienced violence at/near school | 1,697 | 412 | 24.3 |
| Physically hurt near school | 1,697 | 404 | 23.8 |
| Physically hurt by others | 1,697 | 403 | 23.8 |
| Sudden death of someone close | 1,697 | 343 | 20.2 |
| Witnessed or experienced violence within the family | 1,697 | 334 | 19.7 |
| Stressful medical procedure | 1,697 | 321 | 18.9 |
| Threatened, robbed, or exposed to weapon-related violence | 1,697 | 314 | 18.5 |
| Forced/unwanted sexual experience online | 1,697 | 290 | 17.1 |
| Cyberbullying | 1,697 | 274 | 16.2 |
| War-related exposure | 1,697 | 273 | 16.1 |
| Other life event | 1,479 | 259 | 17.5 |
| Forced/unwanted sexual experience | 1,697 | 246 | 14.5 |
| Bullying | 1,697 | 238 | 14.0 |

Supplementary Table S8. Functional impairment prevalence and sensitivity analyses using alternative impairment definitions

**A. Prevalence of impairment across functional domains**

| **Functional domain** | **Yes, n** | **Yes, %** |
| --- | --- | --- |
| Getting along with others | 1,367 | 80.55 |
| Hobbies/Fun | 1,288 | 75.90 |
| School or work | 1,357 | 79.96 |
| Family relationships | 1,313 | 77.37 |
| General happiness | 1,257 | 74.07 |

**B. Distribution of number of impaired functional domains**

| **Number of impaired domains** | **n** | **%** |
| --- | --- | --- |
| 0 | 205 | 12.08 |
| 1 | 43 | 2.53 |
| 2 | 63 | 3.71 |
| 3 | 160 | 9.43 |
| 4 | 197 | 11.61 |
| 5 | 1,029 | 60.64 |

**C. Sensitivity analyses using alternative impairment thresholds**

| **Functional impairment definition** | **Events, n (%)** | **Adjusted PR (95% CI)** | ***p*-value** |
| --- | --- | --- | --- |
| ≥1 impaired domain | 1,492 (87.92) | 1.02 (0.97–1.08) | 0.415 |
| ≥2 impaired domains | 1,449 (85.39) | 1.03 (0.97–1.08) | 0.351 |
| ≥3 impaired domains | 1,386 (81.67) | 1.01 (0.95–1.08) | 0.780 |
| Number of impaired domains† | Mean = 3.88 | 1.00 (0.94–1.07) | 0.916 |

**Note:** Functional impairment was assessed using five CATS-2 questions asking whether the problems reported by participants interfered with (1) getting along with others, (2) hobbies/fun, (3) school or work, (4) family relationships, and (5) general happiness. Responses were coded Yes = 1 and No = 0. The primary definition of functional impairment was endorsement of impairment in ≥1 domain. Therefore, a positive response in a single domain was sufficient for classification. Sensitivity analyses examined thresholds of ≥2 and ≥3 impaired domains. PR = prevalence ratio; CI = confidence interval. †For the number of impaired domains (range 0–5), the effect estimate is an adjusted count ratio rather than a PR. Models compared elevated disaster adversity (≥4 adversities) with low adversity (<4 adversities), adjusted for the prespecified sociodemographic covariates, and used CR2 cluster-robust variance estimation to account for clustering within 20 schools. All analyses included **N = 1,697**.
